# Homes’ Statistical and Pathological Reports on Phthisis Pulmonalis

**Published:** 1838

**Authors:** 


### Art. VII.—Phthisis Pulmonalis.

*Statistical and Pathological Report of the Royal Infirmary of Edinburgh, from* 1833 *to* 1837. By John Home, M. D. President of the Anatomical Society, and Physician to the Northwestern Dispensary.*

There is no disease which has attracted so much attention, of late years, as Phthisis Pulmonalis. Besides the able treatises of Laennec, and Louis of France, of Koch and Englehart of Germany, Gobetti of Italy, Murray and Clark of England, and Morton of our own country, memoirs almost without number have appeared in our medical periodicals, some of them of sterling excellence, others of little or no value. Nor could this be expected to be otherwise. Men enjoy different opportunities and means of improvement; some are rich, and others poor; some have talents and others not; some are industrious, and others indolent; some are practical and others visionary, speculative, and full of "queer imaginings." Emanating from such a diversity of sources, there must necessarily be much that is good, bad, and indifferent, much that is really valuable, and not a little that is contemptible. Of all the lighter and more ephemeral productions, hospital reports

*Edinburgh Medical and Surgical Journal, No. 134.

on whatever subject, are, as a general rule, the most profitable. They are the records of the physical infirmities of the human frame; the chronicles of the nature and progress of diseases, the symptoms which characterise them, the various remedies employed for their cure, their duration, and final termination, accompanied, if the case prove fatal, with an account of the autopsic appearances. Such reports, provided they are drawn up in a plain, concise, and perspicuous manner, have a tendency not only to instruct, but to add materially to our stock of knowledge: they are worth a hundred ordinary dissertations, destitute, as they usually are, of facts, and distorted, as too often happens, with puerile conjectures.

The report of Dr. Home, of which we propose to give a succient analysis, is one of the most interesting that has lately fallen under our notice, and cannot fail to attract largely the attention of the profession. It occupies thirty closely printed pages of our Scotch cotemporary, and there is not a paragraph that does not contain some valuable information. The best way to give our readers an idea of the manner in which the different topics of the report are treated, will be to present to them, in as condensed a form as possible, an account of its principal contents.

The *number* of phthisical patients admitted during the four years and a half, the period embraced by the report, was one hundred. Of these sixty-one were males, thirty-nine females. These results differ widely from those furnished by Dr. Louis and Dr. Clark, in their excellent treatises on consumption, in which it is asserted that the disease is more prevalent amongst women than in men, in the proportion of about one-fifth.

Of ninety-six patients, eleven died before the twentieth year; thirty-one between the twentieth and thirtieth: twenty-nine between the thirtieth and fortieth; seventeen between the fortieth and fiftieth; five between the fiftieth and sixtieth; two between the sixtieth and seventieth; and one between the seventieth and eightieth. From this table it will be seen that the greatest number of deaths in the Edinburgh Infirmary occurred between the ages of twenty and thirty, the next in proportion between thirty and forty, and the next between forty and fifty. These results, as observed by Dr. Home, fully agree with those announced by Dr. Clark, from an examination of the statistical tables of the principal cities of Europe and the United States. "From a review of the above account it would appear that phthisis in Scotland prevails most between the ages of twenty and forty inclusive, which period comprises sixty-six of the cases. Hippocrates fixed the period of its greatest prevalance between eighteen and thirty-five. This appears too early an age for the above cases. Only three of them were between eighteen and twenty; eighteen were between thirty-five and forty. The opinion of Andral, that phthisis is more common among females below the age of twenty, does not appear to be confirmed by the above table. Only five of the females died below that age. The larger proportion of them died after that age. It is remarkable that more females than males died between the ages of forty and fifty, owing probably to the particular change in the female constitution at this period. Andral also found the mortality among women considerable at this period."

No *occupation* was exempt from the disease. Of forty-two persons, whose employments were known, nearly all were mechanics. Masons, painters, and carpenters, formed the most numerous class; probably from their being most exposed to the vicissitudes of the weather, to the inhalation of irritating matter, to intemperance, and to all kinds of dissipation.

The seasons had a considerable influence on the progress and termination of phthisis. It is singular that most of the deaths from this disease should occur in the months of summer, namely, in June, July and August; yet such is the fact, and the circumstance is satisfactorily explained by our author. Most of the cases commence, it is well known, in the winter and spring, and hence, as very few last more than six or eight months, the patient seldom survives the period here specified. Nor is this peculiar to Edinburgh. In Glasgow and Paris the mortality is equally great during summer, as the following table will show.

| Season. | Paris. | Edinburgh. | Glasgow. | Total. |
|---|---|---|---|---|
| Winter | 48 | 40 | 318 | 416 |
| Spring | 54 | 33 | 333 | 420 |
| Summer | 68 | 58 | 361 | 477 |
| Autumn | 64 | 33 | 304 | 401 |
| | 244 | 154 | 1316 | 1714 |

The *duration* of the disease varied in different cases, from a few weeks to several months. Of eighty-seven patients, forty-nine, or considerably more than one-half, died within eight months of the commencement of the disorder. These results agree, in the main, with those of Bayle, Clark, and Louis. The duration was shorter in the female than in the male sex; and in this respect, again, the observations of Dr. Home perfectly coincide with those of Dr. Louis. In a few persons the disease ran its course with great rapidity; and these, it is worthy of remark, were generally considerably advanced in years.

As to the *mortality* in phthisis in comparison with the entire number of deaths, Dr. Home states that it forms a little less than one-seventh, which is very different from the experience of Dr. Louis, who calculated that one half of the patients in the charity hospital of Paris died of consumption. It is to be remembered, however, says the author, that these results are only applicable to phthisis as it occurs in the Royal Infirmary of Edinburgh, not to the complaint generally.

The *symptoms* of phthisis are now so well known that Dr. Home has not deemed it necessary to do more than barely allude to them. Cough, dyspnœa, night sweats, and emaciation, occurred in the greater number of cases : hæmoptysis was witnessed in twenty-six: in almost all, the quantity of blood was small. In four patients only was the hemorrhage considerable, and in two of these it was supposed to arise from the erosion of large vessels. Dropsical symptoms were observed in six—palpitations occurred in four. In ten of the cases there was pain in the epigastric region, with nausea, and occasional vomiting.

We now come to the second division of the report, embracing an account of the pathology of phthisis. The organs of the head in the few cases in which they were inspected, were perfectly healthy. In Dr. Louis' cases, although the intellectual functions were undisturbed, the brain or its envelopes were frequently affected.

Ulcerations of the *larynx* was witnessed only five times, but as the vocal apparatus was not always examined, it is impossible to establish any exact relative proportion of the cases reported by Dr. Home. Nevertheless he feels confident that this organ is much less frequently affected in Scotland than in France, where Louis found it involved in one-fourth of his cases, and Andral still oftener. In nearly all the examples the ulcers were small and superficial, occupying chiefly the epiglottis, the mouth of the larynx, and the vocal cords. The trachea was found ulcerated only in a single case. Occasionally there were patches of redness, generally small, but now and then diffused over a large space. No tubercles were detected. Dr. Home believes these erosions to be peculiar to phthisis, having never observed them in any other diseases, except in syphilis, in which, however, they are much deeper, and attended with more thickening of the surrounding tissues. Their existence during life was indicated by cough, hoarseness, aphony, and pain about the larynx.

The most interesting part of the report is that which relates to the pathological anatomy of the lungs. The essential lesion in pulmonary phthisis, consists, as is well known, in the deposition of tubercular matter. This substance, which occurs either in the form of a hard, greyish, semi-transparent body, or in that of a light yellow, opake, and friable mass, is situated for the most part, Dr. Home thinks, in the air-cells, and in the smaller bronchial tubes. It is on this idea, which is in accordance with that of Carswell and other recent pathologists, that he has founded his division of tubercle into simple, aggregate, and diffused, the foreign matter in the one occupying a single vesicle, several in the second, and in the third one or more lobules, a whole lobe, or an entire lung. Each of these varieties, Dr. Home says, may be subdivided into the *grey* or the *yellow*, according as they consist of the one or the other of the varieties of tubercular substance. This arrangement is certainly unnecessary, as it can answer no useful purpose in practice, and besides is calculated to confuse by its complexity. An outline of it may, nevertheless, be interesting to the reader.

Of the *simple tubercle*, Dr. Home makes two varieties, the grey and the yellow. The former, which corresponds to the miliary granulation of Bayle, and the grey granulation of Laennec and Louis, formed the principal lesion in five of the cases, four of which were males. They occurred between the ages of twenty-two and thirty-nine, were most abundant in the superior lobes, and were observed with nearly equal frequency in both lungs. "These single grey tubercles appear to me," says Dr. Home, "to be the result of a chronic inflammation of the individual air-cells, giving rise to the effusion of a concrete lymph on their lining membranes, which, gradually accumulating from the circumference to the centre, at length fills the whole of their cavities. Occasionally their central portions remain empty, producing the appearance of central depressions, when these tubercles are divided. At other times they contain in their centres a kind of inspissated mucus, giving rise to the appearance, as Dr. Carswell has observed, of central softening so much insisted on by Laennec. More rarely these tubercles contain in their centre bodies derived from without, as grains of sand."

These tubercles may exist in great numbers without giving rise to any symptoms. Percussion generally causes a dull sound if the pulmonary tisse is much condensed, or loaded with seriosity. Occasionally, however, the resonance is nearly natural. From the accompanying bronchitis the stethoscope commonly indicates the existence of different rales. In two of the cases Dr. Home noticed a very peculiar modification of the mucous rhoncus, resembling the creaking of Russia leather, and observable principally during expiration.

The *yellow simple* tubercle is readily distinguished by the peculiarity of its color, and by the softness of its consistence. It usually occurs in groups, in the lungs of children who have died of pectoral affections, supervening upon small-pox, measles, and scarlet fever, the surrounding tissue being generally in a state of red hepatization. This variety of tubercle is supposed by Dr. Home to be the result of acute irritation of the air-cells; and in corroboration of this opinion, he refers to the experiments of Kay, Cruveilheir, and others, in which yellow tubercular matter was commonly produced in the lungs of rabbits by the injection of mercury. When recent it is associated, moreover, with appearances which are the acknowledged effects of inflammation. Adults, it should be remarked, are not exempt from this species of deposit.

The *aggregate tubercle*, again, is divided into two species, the grey and the yellow. They are produced by the filling up of several contiguous air-cells, or the whole of those constituting a lobule, and correspond with the multiple tubercle of Lombard, the agglomerated tubercle of Gendrin, and the tubercular masses of Laennec and Louis.

The grey aggregate tubercle varies in size from that of a pea to that of a walnut, and in color from light grey to nearly black. It is hard, semi-transparent, homogeneous in its structure, and formed, as the name imports, by the agglomeration of a greater or less number of grey simple granulations. When the masses are carefully examined we can sometimes distinguish the different simple tubercles of which they are composed, each of them having a central depression, or a yellowish point. Occasionally they contain particles of silicous matter, as occurred in one of the cases mentioned in the report. This species of tubercle has precisely the same origin, as is supposed by Dr. Home, as the simple grey tubercle previously described.

The *aggregate yellow tubercle*, like the preceding, varies much in size and shape. When recently deposited, says Dr. Home, as we occasionally see it in the lungs of young persons, after attacks of acute inflammation, it often assumes an arborescent or starlike arrangement. When all the cells of a lobule are filled with the morbid deposit, the aggregate mass takes the form of the part in which it is developed. "This is seldom at first round, but angular and lozenge shaped. The round form, I believe, of these tubercles, is afterwards opened when they have been for some time deposited, and is owing to the pressure being equal on all sides. This has the effect, also, of making their texture, which was at first soft and friable, hard and compact. I conceive them to arise from acute inflammation of the lining membranes of several contiguous air-cells, or those of a whole lobule. When soft and recently deposited, they are generally associated with other known effects of inflammation, such as red hepatization of the surrounding pulmonary tissue, and lymph on the neighboring pleura, which, as observed by Dr. Alison, in all respects resembles the matter effused into the substance of the lung."

The third and last kind of tubercle described by Dr. Home is the *diffused*, more generally known since the time of Laennec under the appellation of tubercular infiltration. Like the preceding it consists of two varieties, founded mainly upon the difference of their color, and upon the nature of the inflammatory irritation, which in the one is chronic, in the other acute. The pulmonary structure when thus affected exhibits a homogeneous appearance, and generally cuts like cartilage. In the yellow variety it is moreover, very granular, and seems to be entirely composed of little bodies, which, from the manner in which they are set together, have been aptly compared by Laennec to the eggs of certain insects. These grains are supposed by Dr. Home, and I think very justly, to be nothing more than air-cells filled with yellow matter. The morbid substance, of whatever color it may be, may be limited to several contiguous lobules, or it may occupy a whole lobe, or even the entire organ.

Such is a brief account of the several varieties of tubercular deposites, according to the notions of our author. We have already said that his divisions are objectionable; to make a mere difference of color a ground of distinction, is not only silly, but does not at all comport with the present state of pathological science. For this reason we greatly prefer the arrangement of Laennec, which is now generally adopted by the best writers on the disease before us.

We are glad to find in Dr. Home so strenuous an advocate for the inflammatory origin of tubercular phthisis. Whatever notions may still be entertained upon the subject by some, we have long been convinced that the doctrine in question is the only correct one. Every circumstance, indeed, concerning this morbid deposit conspires to corroborate this view, and it is only surprising that there should ever have been any other.

On the subject of tubercular excavations, Dr. Home has presented us with no new views, and we shall therefore pass it by; referring such of our readers as may be desirous of obtaining fnll information on this point, to the report itself, or to our review of Dr. Louis' work on Phthisis, in the tenth volume of the Western Journal of the Medical and Physical Science.

Adhesions of the opposite surfaces of the *pleuræ* existed in all the cases, generally on both sides, but sometimes only on one.—Their extent was always proportionate to the tubercular state of the lungs, and their strength to the progress of the excavations. The intervening substance was a firm layer, of variable thickness, and usually of a greyish or yellowish color. In sixteen of the cases evidences of acute pleuritis were found. Perforation of the pleura occurred in six, three on the right side, and three on the left. The lesion in most of the cases gave rise to acute inflammation, marked by sudden pain and dyspnœa: in one, death took place in a few hours; in another, in a few days. In several the perforation had existed for some time, and the accompanying inflammation had degenerated into the chronic form. The abnormal opening was in some round or oval, in others irregular and ragged.

The *bronchiæ* were very commonly affected, and it is observed by Dr. Home that few cases of phthisis occur which do not commence with inflammation of their lining membrane. The tubes in one or two instances were dilated. Ulceration was rarely observed; occasionally there was thickening of the mucous tunic.

In regard to the relative frequency of the disease, both lungs were equally affected in twenty-five out of the one hundred cases. In thirty-eight the right lung was most disordered; the left in thirty-seven. Louis in his cases found the left lung much more frequently affected than the right; and Laennec, on the other hand, found the right, the most frequently diseased; so that the result of Dr. Homes' cases holds a sort of middle rank between the experience of these two pathologists.

Out of sixty-six cases in which the *heart* was examined, it was healthy in forty. This is very different, as the author observes, from the experience of Andral, who found the organ diseased in two-thirds of his subjects. The most common affections of the heart were hypertrophy of the ventricles, more frequently of the left than of the right, lesion of the mitral valves, and a patulous state of the oval foramen. Pericardial adhesions and effusions were extremely rare.

The *stomach* was found diseased in nearly one half of the cases, namely, in seventeen out of the thirty-six in which it was specially examined. In seven of the cases it was greatly distended, and altered in its position, extending obliquely downwards as far as the brim of the pelvis. This inordinate dilatation, which Dr. Home believes with Louis to be peculiar to phthisis, was generally accompanied with much paleness and attenuation of the different coats of the organ, as well as in a few instances, with softening. Ulcers were found in two cases; and in one the mucous membrane was thickened and mammillated.

The *small bowel* was examined in sixty-six cases, in more than two-thirds of which it was diseased. In twelve the solitary and agminated glands of the inferior portion of the ileum were enlarged, tuberculated, and of a greyish or yellowish color. Ulcerations were observed in thirty of the cases, or in nearly one half. Louis found them in a much larger proportion in his cases, in seventy-eight out of one hundred and twelve.

The *large bowel,* which was inspected in fifty-five of the cases, was ulcerated in thirty-eight, a proportion considerably greater than in the small intestine. The erosions were generally deep, and occupied a considerable extent of surface. In one instance the bowel was perforated, and the patient died of acute peritonitis. Tubercular matter was rarely seen in the mucous membrane. In one case, in which dysenteric symptoms came on a few days before death, the whole of the large with a considerable portion of the small intestine, was of a red color and covered with recent lymph.

The *mesenteric glands* were enlarged in eight of the cases; but as they were seldom examined nothing can be said about the relative frequency of their diseases. Three of the cases occurred in children, in whom they produced symptoms of *tabes mesenterica,* masking in a great measure the phthisical. In adults they produced no symptoms.

The *liver* was very commonly diseased. Out of seventy-five cases in which it was examined, it was found healthy only in fourteen. In twenty-three it exhibited different forms of the early stages of *cirrhosis,* in which the organ becomes enlarged, indurated, and variegated in color like the section of a nutmeg, with dark red spots scattered over a light yellow ground. Neither Louis nor Andral take notice of such an affection of the liver in their dissections of phthisical subjects. They mention the *fatty* state of the organ as having occurred in one third of their cases. In Dr. Home's examinations it was observed in a smaller proportion, in ten out of sixty-five persons. The organ was of a light reddish yellow color, dotted over with brownish spots, soft in consistence, and quite unctuous to the feel. This lesion occurred more frequently in women than in men, in the proportion of nine to one. Many of the individuals in whom it was seen, had been addicted to the use of intoxicating liquors.

The liver had occasionally a *waxy* appearance. This change, which Professor Mayo has described under the name of *steatosis,* and which is probably only an advanced stage of the fatty degeneration, occurred in five cases, four females and one man. The liver was often very much enlarged; and in three instances it contained tubercles.

The *spleen* was rarely much diseased. It contained tubercles in two cases: in a third, there was considerable thickening of its capsule; and in a fourth, a piece of bone, about the size and shape of the patella, was embedded in its substance.

The *kidneys* were almost always healthy. In four of the cases they had undergone the granular degeneration first noticed by Dr. Bright of England: in two others they were unusually pale: in none were there any tubercles found.

The *peritoneum*, on the whole, was not often diseased. Serum was found in its cavity in three cases, in two of which it was associated with dropsical effusions in other parts of the body.—Lymph and water, the result of acute inflammation of the membrane, were observed in two instances; in the one, in which it was general, it was evidently caused by a perforating ulcer of the colon; in the other it was more partial, being confined to the right iliac region.

Such is an outline of the report of Dr. Home. We ask our readers to give it an attentive perusal; persuaded that they will be both interested and instructed by it. Dr. Home has laid the profession under deep obligations, both in this country and in Europe; and humble as the task certainly is, we shall always be happy to analyse his productions, especially when they possess such sterling merit as the one here noticed. S. D. G.

